# Chloride of Sodium in Intermittent Fever

**Published:** 1852-09

**Authors:** 


*Chloride of Sodium in Intermittent Fever.*—Wishing, then, to experiment with salt, a few cases of intermittent fever (old stagers), contracted in Algiers were selected as subjects. Behold, then, Piorry at the bedside. The patient asserts that he contracted the fever and ague several years since in Africa; that he has frequently been cured; but that the disease has constantly reappeared at the end of fifteen days or one month at farthest. The type of the fever is tertian. The spleen is percussed and found to be abnormally dull throughout its whole extent; the entire splenic region is sensitive upon percussion, particularly over the dullest points; and each blow is accompanied by marked contortions of the countenance. This sensibility extends but little beyond the region of dullness, which last occupies an extent of fifty-three lines, measuring in the direction indicated above. To this patient a drachm of salicine is administered without producing any change in the dimensions of the spleen. A few minutes subsequently, half an ounce of salt mixed with a cup of soup is given, and upon carefully percussing the splenic region at the end of four minutes, this organ is found diminished one inch, from above downwards. The next day the spleen is found to be of the same size, but upon the administration of a second dose of salt, it suddenly contracts and measures nearly three quarters of an inch less than yesterday. The resonance throughout the entire organ has increased while the sensibility has diminished. The succeeding day, the attack of fever is very slight, and upon giving a third dose, the disease does not return; and when seen six weeks subsequently, the patient is still free from his African enemy. Thus we see that a diminution of twenty four lines in the length of the spleen was the result of the medicament, the fever being cured more effectually than ever before; *i. e.*, the patient had remained free from all relapse for the space of six weeks; one month having previously been the longest period of immunity.

We have the notes of seven cases of well-marked intermittent fever, in all of which the administration of the chloride of sodium was followed by rapid decrease in the volume of the spleen and cure of the febrile symptoms. We also have the record of three cases in which salt was unsuccessfully used; in one of these, the sulphate of quinine effected a cure; in a second it too failed, while in the third it was not tried. These were all well-marked cases of intermittent fever, such as would pass muster in any of our own malarious districts.

Let it be remembered that most of the fever and ague met with in the Parisian hospitals, is of long standing, and imported from the malarious districts of Algiers, which generate a form of the disease even worse than that found amid the marshes on the banks of the famed Maumee; that these cases have been treated again and again, have been cured now by the sulphate of quinine, now by arsenic, but only to reappear upon the slightest exposure or imprudence; in short, to recur as only *the shakes* can recur.

We witnessed many of the experiments of M. Piorry, and in the great majority of them, the fever yielded to salt quite as readily as to the salts of quinia. And as to the theory of M. Piorry, the spleen diminished under the use of the remedy, *pari passu*, with the febrile symptoms, in every case where the disease was cured, proving that this organ really shows the influence of remedies over this class of fevers—that it is, as it were, a febro-barometer —for the diminution of the spleen is a constant phenomenon accompanying the cure of the disease, whatever be the curative agent employed.

M. Piorry's method of administering the chloride of sodium is, to give half an ounce in a cup of thin soup during the apyrexia and fasting. It usually agrees with the stomach perfectly well, but in some few cases we have seen it excite vomiting and diarrhea. Three doses commonly suffice to effect a cure, the first two to be taken on succeeding days, and the third after an interval of one day. Should the spleen be undiminished in volume by the first dose, we may be sure that the remedy will not cure the disease; and the same is true of all the antiperiodics. Excepting in rare cases, the diminution of the spleen occurs immediately upon the administration of the remedy (salt or sulph. quinine,) and may frequently be detected within one minute, after which the organ remains stationary until a second dose of the medicament be administered.

Is the chloride of sodium as efficient an antiperiodic as the sulphate of quinine? Are the cures effected by the one as permanent as those effected by the other? The first question can only be answered by those possessing a larger field of observation than the writer. May we not hope for a solution from those of our profession who observe the disease too largely either for comfort or pleasure? In regard to the permanency of the cures, we apprehend there is not much difference, be the medication what it may; for relapses are only too common after the greatest care and most patient attention.

Should the discovery prove as useful and applicable as it promises, the benefit accruing from it will be immense. If it be capable of taking the place of the sulphate of quinine in the majority, or even in one half the cases of intermittent fever, therapeutics will be largely the gainer.—*Ibid.*

